# Exploring the Clinical Potential of Dynamic Digital Radiography: A Narrative Review

**DOI:** 10.1002/jmrs.70060

**Published:** 2026-02-04

**Authors:** Connor W. Braniff, Mohamed K. Badawy

**Affiliations:** ^1^ Monash Health Imaging Monash Health Clayton Victoria Australia; ^2^ Department of Medical Imaging and Radiation Sciences, School of Primary and Allied Health Care, Faculty of Medicine, Nursing and Health Sciences Monash University Clayton Victoria Australia

## Abstract

Dynamic Digital Radiography (DDR) is an emerging X‐ray technology that captures anatomical motion through rapid sequential imaging, typically at 15 frames per second. By enabling real‐time visualisation of physiological processes, DDR offers functional insights that extend beyond the capabilities of traditional static radiography. This literature review evaluates current evidence on DDR's clinical applications, with particular attention to pulmonary assessment, cardiac motion analysis and orthopaedic evaluations. Recent advances in post‐processing software, including motion quantification and enhanced visual analytics, further support DDR's potential utility in a range of clinical settings. Findings from existing studies indicate that DDR provides meaningful functional information that may assist clinicians in evaluating disease progression, monitoring treatment response and identifying subtle dynamic abnormalities. Its portability and relatively low radiation dose are notable advantages, especially for patients with limited mobility or for point‐of‐care use. DDR has shown diagnostic performance comparable to established modalities such as CT, conventional radiography and fluoroscopy in selected applications, suggesting value as a complementary imaging tool. Nevertheless, the review also highlights important limitations. Current research is constrained by small study populations and limited long‐term data. Although DDR offers versatility and unique dynamic imaging capability, its ability to outperform established modalities is unclear, making its suitability as a replacement in routine clinical practice uncertain. Broader adoption will require further large‐scale, standardised studies to establish diagnostic accuracy, reproducibility and cost‐effectiveness. Overall, DDR represents a promising adjunct to existing imaging technologies. Its ability to capture real‐time anatomical motion positions it as a valuable emerging tool with the potential to enhance functional assessment and expand imaging accessibility across varied clinical environments.

## Graphical abstract

Dynamic Digital Radiography (DDR) is a promising X‐ray technology that captures rapid image sequences, enabling the visualisation of moving anatomical structures for various clinical applications, including pulmonary, cardiac and orthopaedic assessments. While DDR offers benefits like portability and post‐processing software, its potential to replace traditional imaging methods such as CT, X‐ray and fluoroscopy remains uncertain due to challenges in consistent performance and clinical adoption.

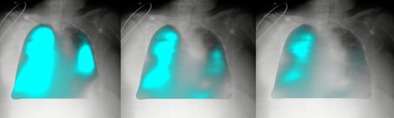

## 1 Introduction

Dynamic Digital Radiography (DDR), or serial radiography, represents a recent advancement in X‐ray imaging technology, allowing for the observation of anatomical structures in motion. Developed in the early 2000s, DDR has garnered increased attention due to recent enhancements in hardware and image processing software, enabling automatic segmentation and identification of moving anatomical structures, such as the diaphragm and lungs [1, 2]. Unlike traditional static radiographic images, DDR captures sequences of images at up to 15 frames per second, providing clinicians with a dynamic portrayal of physiological and pathological conditions that are not visible in standard projectional radiography [3].

The acquisition process for DDR closely mirrors that of conventional radiography, requiring minimal additional training for radiographers. This makes DDR an efficient imaging modality, offering both static and dynamic imaging capabilities with only a slight increase in acquisition time compared to standard radiography [1, 4]. Furthermore, DDR has been identified as a cost‐effective alternative to more complex imaging systems like Computed Tomography (CT) and fluoroscopy, particularly regarding radiation dose. While CT involves multiple exposures for cross‐sectional imaging and continuous fluoroscopy results in higher doses due to its real‐time nature, DDR delivers a significantly lower radiation dose, making it a safer option for patients [3].

Despite both modalities providing moving images, it is crucial to distinguish DDR from fluoroscopy. DDR offers a larger field of view and lower radiation exposure than continuous fluoroscopy [4]. DDR still maintains a dose advantage compared to pulsed fluoroscopy, which reduces radiation dose using short pulses. Studies indicate that DDR can deliver only 10% of the radiation dose associated with conventional fluoroscopy in specific applications, such as chest exams [5, 6, 7].

As DDR is a rapidly evolving imaging modality, regular updates are warranted to capture its expanding applications and technological advancements. This narrative review extends beyond prior works by providing an updated analysis of DDR's use in both clinical and experimental settings. It aims to explore DDR's role as both a fixed and mobile imaging modality, highlighting its evolving applications and potential impact on clinical practice.

## 2 Method

This narrative review examines the clinical and experimental applications of DDR. The primary literature search was performed using Embase (via OVID), PubMed and Scopus, with the libraries selected due to their comprehensive indexing of biomedical and clinical research, particularly in radiology and imaging sciences. The search was limited to studies published between January 2010 and October 2024, aligning with the period of DDR's active clinical implementation, with the following keywords utilised: ‘Dynamic Digital Radiography’, and ‘Dynamic Digital X‐ray’.

To enhance the scope of the search, the search was supplemented by a curated bibliography provided by KONICA MINOLTA, which identified additional relevant studies not captured in the initial database search. This bibliography included peer‐reviewed publications and was used to ensure that studies potentially missed due to variations in terminology or indexing were considered. Such supplementary material is particularly valuable in emerging fields like DDR, where inconsistent or proprietary terminology may limit the effectiveness of conventional database searches.

**FIGURE 1:**
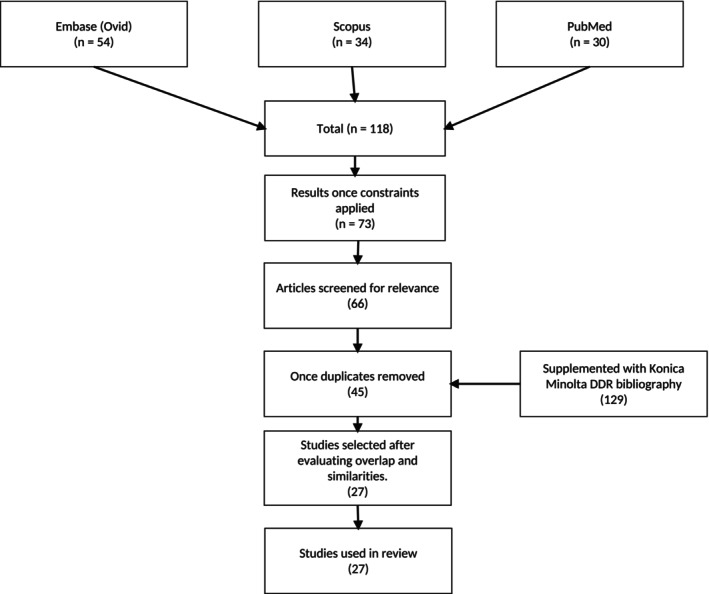
Literature identification and screening process.

All identified studies were screened to ensure inclusion only of those in which DDR was the primary focus, with particular emphasis on studies investigating clinical or potential diagnostic applications. Studies that were not directly related to DDR's diagnostic relevance, duplicate or overlapping studies, those published outside the specified timeframe, or those not available in English were excluded. It is acknowledged that the use of focused search terms may have excluded studies that referred to DDR using less common or manufacturer‐specific terminology, which can occur in a niche and emerging field where terminology is not yet standardised. Figure 1 provides an overview of the literature screening process.

## 3 Clinical Implementation and Technological Advances

Dynamic Digital Radiography (DDR) represents a significant evolution in radiographic imaging by enabling the acquisition of dynamic, real‐time images that capture anatomical motion and interactions, a capability beyond the static snapshots provided by traditional radiography [8]. This flexibility allows DDR to image patients in multiple positions, including standing, seated, or supine, broadening its clinical applicability across various patient populations and conditions. Early adoption of DDR was hindered by technological limitations, such as lack of portability and unclear cost–benefit compared to established imaging modalities like computed tomography (CT), magnetic resonance imaging (MRI) and fluoroscopy, which were already well entrenched in clinical practice [1]. However, recent advancements have addressed these issues. Notably, the development of portable DDR systems, which leverage mobile X‐ray technology combined with post‐processing software such as Motion Analysis Workstations (e.g., DI‐XI), has expanded DDR's utility, particularly for bedside imaging of immobile or critically ill patients [1]. These technological improvements have contributed to a growing body of clinical research and increased DDR adoption, as summarised in Table 1.

**TABLE 1:** Summary of literature concerning clinical implementation of Dynamic Digital Radiography.

Publication	Aim	Method	Cohort	Dose (mGy)	Acquisition parameters	Key findings	Level of evidence
Hida et al. (2019) [9]	Assess diaphragmatic motion during forced breathing by time‐resolved quantitative analysis using DDR explorimg associations between diaphragmatic motion and participants' demographics and pulmonary function	Volunteers underwent DDR during forced breathing in a standing position, with automatic tracking of diaphragm positioRns and motion metrics. Statistical analyses were performed to evaluate the relationship between diaphragm motion, demographics and pulmonary function	174	0.3–1.0	100 kV, 50 mA, 15 fps, 17–37 s exposure	DDR effectively demonstrated the characteristics of diaphragmatic motion during forced breathing	Research Article
FitzMaurice et al. (2021) [10]	To explore the utility of DDR as a tool to assess suspected diaphragm palsy	DDR was used to assess patients with suspected diaphragm palsy. Diaphragm position and velocity were calculated for comparison with fluoroscopy	8	< 0.125	15 fps, 10–19 s exposure	DDR successfully identified paradoxical diaphragm motion in 6 of 8 cases and abnormal motion in the remaining 2, consistent with fluoroscopy results	Abstract
Uchida et al. (2024) [11]	Assessment of DDR's implementation in the surgical treatment of diaphragmatic paralysis	DDR was utilised for preoperative imaging to identify diaphragmatic paralysis with paradoxical movement, and postoperatively to demonstrate improvement following surgical intervention	1	1.7	Not reported	DDR enabled real‐time, quantitative assessment of bilateral diaphragmatic motion, allowing effective evaluation of respiratory movement	Case Report
FitzMaurice et al. (2022) [12]	Assess the utility of DDR in assessing diaphragm motion in patients with suspected diaphragm dysfunction	Patients referred for the assessment of diaphragm function due to clinical indications underwent DDR with voluntary sniff manoeuvres	21	0.25	20s exposure	DDR identified paradoxical diaphragm motion in 14 patients, and reduced excursion in 4. DDR warrants further investigation in assessment of diaphragm dysfunction	Pilot Study
Shibuya et al. (2022) [13]	Assess DDR's ability to effectively detect Bilateral recurrent laryngeal nerve paralysis (BRLNP)	DDR was performed as a screening test in two clinical case‐reports to diagnosis BRLNP	2	1.16	Not reported	DDR demonstrated a clinically useful and non‐invasive screening test for accurate diagnosis of BRLNP	Case Report
Koyama et al. (2024) [14]	Assessment of DDR in bedside diagnosis of silent aspiration by examining default grayscale images (DGI) and inverted grayscale images (IGI)	Participants referred from primary physicians for swallow assessments. Using DDR DGI and IGI images were taken and assessed for level of agreement to penetration‐aspiration scale (PAS, the gold standard for diagnosis)	18	Not reported	60 kV, 80 mA, 15 fps	IGI and DGI showed 100% accuracy for normal range, 80–100% for penetration and 83–100% for aspiration, with near‐complete agreement between methods for abnormal findings	Preliminary exploratory Study
Fyles et al. (2023) [15]	Evaluating DDR as a low‐dose method for detecting large airway collapse	A 56‐year old under‐went both CT with inspiratory/expiratory slices and DDR during tidal and forced breathing, with tracheal diameter measured and compared between modalities	1	Not reported	12 s exposure	DDR showed significant tracheal narrowing from 13.6 to 3.1 mm during forced expiration, corroborating CT findings but with a much lower radiation exposure	Case Report
Ohkura et al. (2021) [16]	Identify the relationship between parameters obtained from DDR and ventilatory disorders	DDR was used to obtain sequential chest images of a patients taking slow and maximum breathing, with various ventilatory assessments taken	273	0.2	100 kV, 50 mA, 15 fps, 14 s exposure	Patients with obstructive disorders and restrictive disorders showed decreased diaphragmatic motion and lung area change rate, showing potential for the evaluation of ventilatory disorders	Research Article
Ohkura et al. (2024) [17]	To evaluate the accuracy of DDR in the assessment of pulmonary function compared to conventional pulmonary function tests (PFTs)	Pulmonary function was assessed using both conventional PFTs and DDR imaging based on slow vital capacity, with results compared using correlation and regression analyses	102	Not reported	Not reported	DDR showed strong correlation to PFTs in estimating vital capacity (*r* = 0.87) and forced expiration volume (*r* = 0.76). DDR demonstrated high sensitivity for detecting obstructive disorders (96.7%), but low specificity (18.6%). The sensitivity and specificity for detecting restrictive disorders was low (11.1%) and high (97.6%) respectively	Abstract
Kitahara et al. (2019) [18]	Development of deep learning‐based lung segmentation for DDR	A convolution neural network was trained (300 images) and tested (234 images) from images produced from DDR, with post‐processing applied to reduce segmentation errors and assess lung area changes across respiratory phases	214	Not reported	100 kV, 0.2 mAs, 15 fps, 10s exposure	The model achieved high segmentation accuracy (DICE of 0.94–0.95), and analysis of lung area changes proved useful for detecting obstructive pulmonary patterns	Abstract/Conference Paper
Ishihara et al. (2021) [19]	To estimate respiratory changes in lung volumes using frontal and lateral DDR	XCAT phantoms representing different sexes and body types, where used to train a CNN, which was then used to estimate lung volumes in DDR images	20	Not reported	Not reported	CNN‐based approach successfully estimates lung volumes from frontal and lateral DDR with strong correlation. DDR shows potential for estimating lung volume, corresponding to vital capacity and pulmonary function tests	Abstract
Watanabe et al. (2023) [20]	To evaluate the utility of DDR for pre‐operative detection of pleural adhesion to aid surgical planning	Participants were imaged with DDR prior to surgery, with pre‐operative evaluation using three imaging analysis modes	120	1.7	100 kV, 80 mA, 15 fps, 14 s exposure	Accurate pre‐operative evaluation of pleural adhesion was confirmed in 84.9% of patients with a sensitivity of 64.5%, specificity of 91%, positive predictive value of 74.1% and negative predictive value of 88.0%	Research Article
Nikaido et al. (2024) [21]	Explore DDR application as a predictor of disease progression in idiopathic pulmonary fibrosis (IPF)	Two IPF patients underwent DDR to assess diaphragmatic dynamics, which were correlated with pulmonary function and clinical progression	2	Not reported	80 kV, 56 mA, 15 fps, 20s exposure	DDR demonstrated promise in reflecting disease progression in IPF, suggesting a non‐invasive, low‐dose modality for pathophysiological evaluation	Case Report
Ueyama et al. (2021) [22]	Evaluation of DDR's ability to predict forced vital capacity (FVC) and other pulmonary function tests in interstitial lung disease (ILD) patients	ILD patients had lung field areas measured using DDR, allowing lung volumes at maximum inspiration and expiration to be estimated. Correlation coefficients between measured values from DDR and PFT parameters were calculated	97	PA: 1.5 LAT: 4	PA: 100 kV, 80 mA LAT: 120 kV, 100 mA, 15 fps, 19 s exposure	DDR lung volumes showed strong correlation with FVC (*r* = 0.86). Interclass correlation coefficients between measurements of inspiration and expiration were 0.94 and 0.88, respectively (*p* < 0.001). A multi‐linear model for predicting FVC based on various parameters was also developed, demonstrating high accuracy ($R^{2}$ = 0.814)	Research Article
Tanaka et al. (2021) [23]	Assessment of ventilation and perfusion metrics derived from DDR imaging	Ventilation and perfusion metrics obtained using DDR from changes in lung density across subdivided lung regions were compared to radioactive agent uptake from nuclear medicine scans	42	< 1.9	100 kV, 0.2 mAs/pulse, 15 fps, 14 s exposure	Moderate correlation was found between perfusion and ventilation metrics obtained through DDR and radioactive agents (*r* = 0.57 and 0.53, respectively, *p* < 0.001). Good to strong correlation was found in 80.9% of patients for perfusion metrics and 52.4% in ventilation metrics (*r* = 0.60 and 0.53, respectively). Dice similarity coefficients indicated moderate correlation for both metrics	Research Article
Ohkura et al. (2020) [24]	Determine the utility of DDR for pulmonary function assessment in patients with airflow limitations	Patients with airflow limitations were imaged with DDR, with the relationship between lung area and rate of change in lung area due to respiratory motion with respect to pulmonary function was assessed	118	0.21	100 kV, 50 mA, 15 fps, 14 s exposure	Rate of change in lung area for maximum inspiration and expiration was associated with the residual volume to Total lung capacity ratio (*r* = 0.48, *p* < 0.01) and the percentage of predicted FEV (*r* = −0/33, *p* < 0.01) in patients with limited airflow	Research Article
Hanaoka et al. (2021) [25]	To evaluate whether dynamic perfusion DDR can accurately predict postoperative pulmonary function and complications in patients undergoing radical lung cancer resection	DDR, pulmonary perfusion scintigraphy and spirometry were performed before and after surgery in patients, and correlations between predicted and measured pulmonary function parameters were assessed	52	< 1.5	100 kV, 40 m, 10 s exposure	DDR showed excellent correlation with scintigraphy in predicting postoperative FEV_1_ and diffusion capacity for carbon monoxide (*r* ≈ 0.88–0.92), accurately identifying patients at risk of respiratory complications and demonstrating its potential as a simpler, cost‐effective alternative for preoperative functional assessment	Research Article
Santibanez et al. (2024) [26]	To develop and compare DDR derived pulmonary function tests (dPFTs) and conventional PFT measurements	A CNN was developed capable of quantifying lung areas in DDR was used to generate dPFT's, which was compared to PFT's with patients experiencing normal, obstructive and restrictive lung physiology	55	0.3–1.0	Not reported	Strong correlation was observed between dPFT and PFT, including total lung capacity (*r* = 0.764), FEV (*r* = 0.591), vital capacity (*r* = 0.763) and functional residual capacity (*r* = 0.756) all for a *p* value of < 0.000001	Research Article
Hoshino et al. (2021) [27]	To evaluate the utility of DDR in the assessment of left–right pulmonary blood flow differences in patient with congenital heart disease	DDR was used for a postoperative patient with partial anomalous pulmonary venous connection, where PH2 mode was used to measure pulmonary blood flow ratios, which was compared to ventilation/perfusion scintigraphy	1	< 1.9	Not reported	Scintigraphy showed no difference in ventilation between the right and left sides, with a blood flow ratio of 64.4:36.6. PH2 showed similar results to that produced by scintigraphy with blood flow ratios of 75.6:24.4. DDR was determined to be superior due to its dynamic evaluation method with less radiation exposure	Case Report
Toyomura et al. (2023) [28]	Evaluation of DDR for pulmonary blood flow assessment in a patient with congenital uni‐lateral absence of the pulmonary artery	DDR was used to quantify lung perfusion in a patient with right pulmonary artery graft stenosis, which was compared to conventional lung perfusion scintigraphy	1	Not reported	Not reported	Tc‐99 m lung perfusion showed decreased perfusion in the right lung (right/left: 20.1%/79.9%). A similar result was achieved with DDR (17.4%/82.6%)	Case Report
Yamasaki et al. (2023) [29]	To compare the performance of DDR and lung V/Q scanning for the detection of chronic thromboembolic pulmonary hypertension (CTEPH)	A retrospective analysis of patients with pulmonary hypertension who underwent DDR and V/Q scanning was conducted, with CTEPH diagnosis confirmed with pulmonary angiography	50	1.6	85–95 kV, 250 mA, 15 fps, 7–10 s exposure	Observe tests indicated the sensitivity, specificity and accuracy of DDR was 97%, 86% and 92%, respectively. Area under the receiver operating characteristic curve for DDR was 0.92, with agreement between the consensus interpretation of DDR and V/Q screening substantial (κ = 0.79)	Research Article
Yamasaki et al. (2024) [30]	To assess the principles, advantages and clinical applications of DDR for pulmonary vascular diseases	A review was conducted of existing knowledge on DDR and its clinical applications	Not reported	0.2	15 fps, 7–15 s exposure	DDR is a novel imaging technique that demonstrates pulmonary perfusion, with almost no contraindications. Image findings suggest DDR are highly correlated with anterior images of planar perfusion scintigraphy, coronal view or iodine map of CTPA, and invasive pulmonary angiography	Review Article
Kitamura et al. (2022) [31]	To demonstrate the usefulness of DDR and Kinovea to assess lung tumour motion	DDR and a respiratory‐gated 4D CT were performed on patients undergoing stereotactic body radiation therapy for lung tumours. Kinovea was used on DDR to track tunour motion	15	PA: 5 LAT: 6.4	PA: 85 kV, 80 mA LAT: 120 kV, 100 mA 15 fps, 20 s exposure	Spearman's rank correlation and Wilcoxon signed‐rank tests showed significant correlation between the amplitude of tumour motion between DDR and D4‐CT in SI and LR directions and SRSS (*r* = 0.95, *p* < 0.001, *r* = 0.70, *p* = 0.004, and *r* = 0.92, *p* < 0.001, respectively), while DDR median amplitude was significantly higher than 4D‐CT in all directions	Research Article
Hiraiwa et al. (2023) [32]	To assess DDR;s ability to estimate heart failure hemodynamics	Heart failure patients underwent DDR images and right heart catheterisation. Pixel value changes obtained from DDR were measured and correlated with invasive hemodynamic parameters	20	1.8	Not reported	Significant correlation was observed between mean right atrial pixel value and atrial pressure (*r* = −0.576, *p* = 0.008), mean right pulmonary artery pixel value and pressure (*r* = −0.546, *p* = 0.001), left ventricular apex pixel value change rate with atery wedge pressure (*r* = −0.664, *p* = 0.001), and cardiac index (*r* = 0.606, *p* = 0.005)	Research Article
Hussian et al. (2023) [33]	To assess DDR as a novel imaging technique for assessing posterior shoulder instability	A case report was conducted on a 28‐year‐old male with recurrent posterior shoulder dislocations, with DDR utilised to assess the humeral head and glenoid under stress during range of motion	1	1.33	Pulsed radiographs 15 Hz for up to 20 s	DDR effectively demonstrated posterior humeral head subluxation and dynamic reduction during movement, offering real‐time assessment of instability. Enabling a sophisticated assessment of anatomical structures	Case Report
Gezici et al. (2017) [34]	Evaluate non‐surgical versus surgical treatment in elderly patients with upper cervical spinal injuries	Patients within this study were monitored using DDR	16	Not reported	Not reported	DDR appeared to provide a sufficient imaging modality for the retrospectively assessment of the upper cervical spinal injury patients	Research Article
Sakuda et al. (2014) [35]	To develop a functional form of radiography to perform a quantitative analysis for the shoulder joint	Dynamic images were obtained to evaluate functional movement of the shoulder joint	8	0.4	90 kV, 200 mA, 3.75 fps, 4 s exposure	Dynamic radiography allowed a low dose evaluation of joint motion	Research Article
Grey et al. (2019) [36]	To characterise three‐dimensional joint kinematics during natural overground walking	Dynamic X‐ray imaging was used to measure 3D motion of the entire knee‐joint complex, femur, tibia and patella, when humans walked over ground at natural speed	15	Not reported	110 kV, 13.1 mA, 200 fps, 1/200 s exposure	Dynamic X‐rays were able to provide an appropriate modality to assess knee‐joint motion while a patient was walking	Research Article

## 4 Radiation Dose Considerations

Radiation exposure remains a critical consideration when integrating new imaging modalities into clinical practice, especially for procedures necessitating repeated or serial imaging. DDR has demonstrated favourable radiation dose profiles, generally lower than many conventional imaging techniques. Fyles et al. reported median entrance surface doses around 0.65 mGy for fixed DDR systems, with lateral images requiring higher doses, up to 4 mGy, due to increased tissue thickness and the need for greater X‐ray intensity to achieve adequate penetration [2]. The effective radiation dose for DDR approximates 0.2 mSv, roughly equivalent to the dose from five standard posterior–anterior and lateral chest X‐rays, and significantly lower, by a factor of approximately 44, than a chest CT scan without contrast [37, 38]. This reduced dose profile supports DDR's suitability for applications requiring frequent imaging, such as monitoring of respiratory function or pulmonary conditions, where minimising cumulative radiation exposure is essential.

## 5 Thoracic Imaging: Diaphragm and Airway Function

**FIGURE 2:**
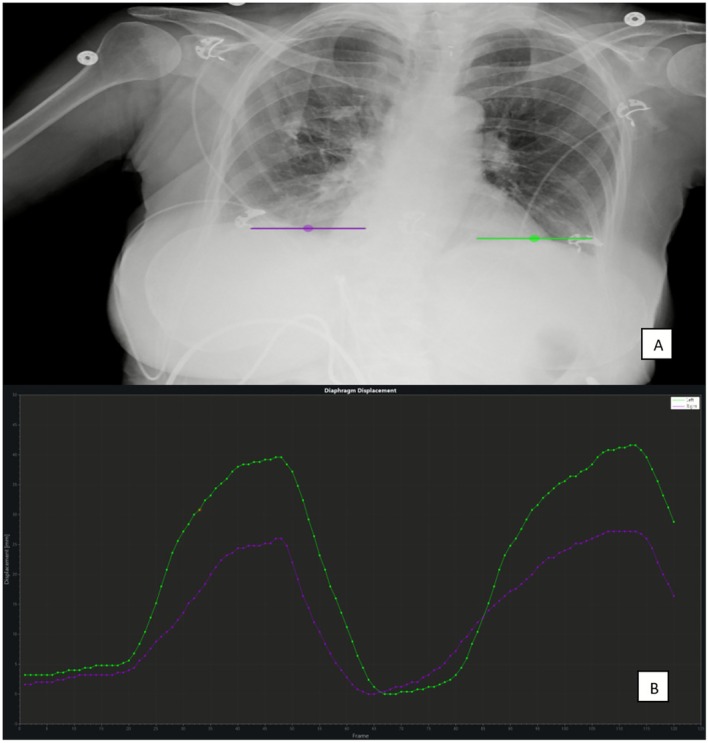
Quantification of diaphragm motion through the utilisation of a motion time graphs (B) of defined points on an original DDR acquisition (A). (Images provided by Minolta Healthcare Americas, used with permission. Obtained from a mobile DDR system in an ICU.)

DDR offers a novel approach to thoracic imaging by enabling dynamic visualisation and quantification of diaphragm motion, which is challenging to capture accurately with static chest radiographs. Traditional methods typically provide only static assessments, often missing subtle or dynamic abnormalities. DDR's capacity to generate motion‐time graphs, illustrated in Figure 2, and quantify parameters such as diaphragmatic excursion and velocity facilitates the diagnosis of conditions like diaphragm palsy and hemidiaphragm dysfunction [1, 9, 10, 11, 12]. For instance, studies have documented significant quantitative differences in diaphragm motion and lung area between affected and unaffected sides, reinforcing the clinical utility of DDR in respiratory function evaluation [12]. These findings highlight DDR's potential to improve diagnostic accuracy for respiratory conditions that are otherwise difficult to detect using conventional imaging.

**FIGURE 3:**
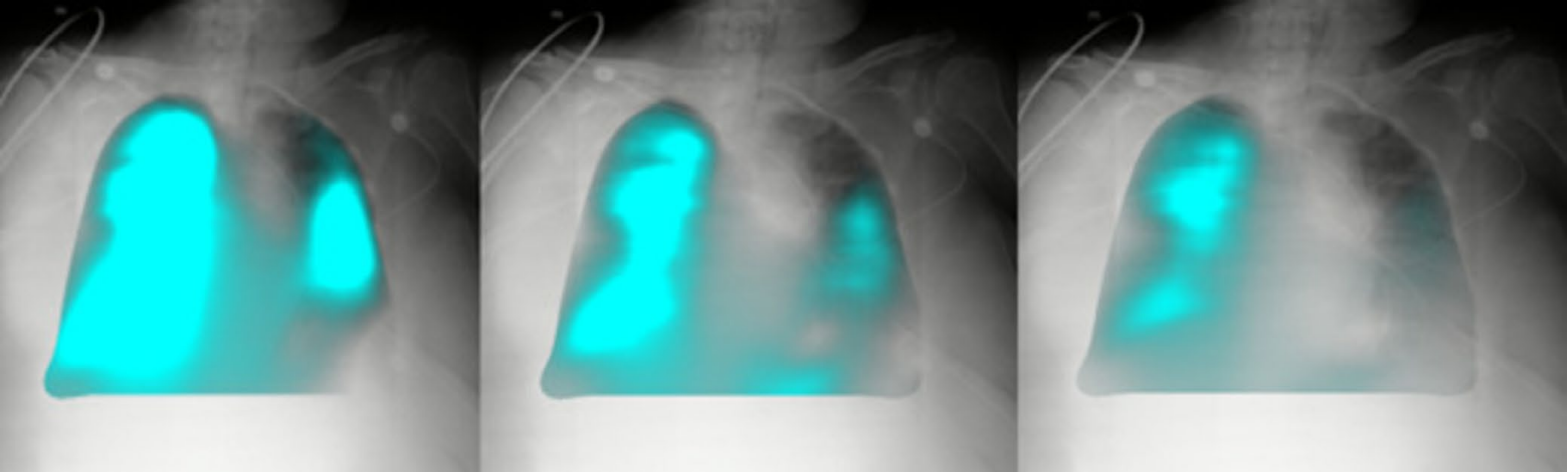
Visualisation of pixel change associated with respiration over a breathing cycle provided by PL‐Mode from a DDR acquisition. (Images provided by Minolta Healthcare Americas, used with permission. Obtained from a mobile DDR system in an ICU.)

Beyond diaphragm assessment, DDR has been applied effectively to evaluate airway function and lung ventilation. Using specialised post‐processing software modes such as PL‐Mode, depicted in Figure 3, DDR visualises changes in lung signal intensity corresponding to respiratory cycles, thereby facilitating assessment of ventilation dynamics [13]. Clinical applications of DDR in this domain include the diagnosis of bilateral recurrent laryngeal nerve paralysis, typically identified with flexible laryngoscopy but now accessible through DDR imaging with lower radiation exposure and improved patient comfort [13]. DDR has also shown promise in assessing silent aspiration in patients with dysphagia, particularly those unable to undergo fluoroscopic swallowing studies due to limited mobility. Diagnostic accuracy rates for DDR in aspiration assessment exceed 80%, with strong concordance to conventional videofluoroscopic techniques, suggesting DDR as a reliable, less invasive alternative [14].

Additionally, DDR serves as a low‐dose imaging option for detecting large airway collapse, providing comparable results to CT imaging while reducing radiation burden. However, further validation is needed to address variability in patient conditions that may affect diagnostic consistency [15]. Research correlating DDR‐derived parameters with standard pulmonary function tests (PFTs) supports DDR's role as a complementary diagnostic tool in respiratory medicine. Moderate to strong correlations have been observed between DDR metrics (e.g., lung area, tracheal diameter, diaphragmatic motion) and conventional PFT values such as vital capacity and forced expiratory volume (FEV), indicating DDR's potential for pulmonary function assessment [16, 17]. Integration of deep learning techniques has further enhanced DDR's utility, enabling accurate lung segmentation and volume estimation from DDR images, which may improve quantitative analyses and support automated or semi‐automated diagnostic workflows [18, 19].

## 6 Pulmonary Function and Disease Monitoring

DDR has shown promise in detecting pleural adhesions and monitoring interstitial lung diseases. For example, Watanabe et al. reported DDR diagnosing pleural adhesions with 84.9% accuracy, though further improvements are needed to surpass ultrasound as the preferred modality [20]. DDR can track idiopathic pulmonary fibrosis (IPF) progression by analysing respiratory cycles and diaphragmatic motion, distinguishing stable from progressive cases [21]. Similarly, Ueyama et al. demonstrated strong correlations between DDR‐derived parameters and forced vital capacity (FVC) in interstitial lung disease patients, highlighting DDR's non‐invasive diagnostic potential [22].

Furthermore, DDR can assess ventilation and perfusion metrics by comparing radiographic lung density changes with nuclear medicine imaging, showing moderate correlations (*r* ~ 0.5) that support DDR as a non‐invasive lung function tool [23]. N. Ohkura et al. examined air trapping patterns through lung area changes in DDR images and investigated correlations with pulmonary function metrics. For the FEV, while the correlation was statistically significant (*p* < 0.01), the Pearson correlation of −0.33 is generally considered weak. This suggests that while there is a relationship between the change in lung area and the predicted FEV, the strength of the relationship isn't strong, and this study should be further evaluated to validate the clinical relevance of the association between DDR and FEV [24].

In addition, Hanaoka et al. demonstrated that DDR‐derived blood flow ratios showed excellent correlation with pulmonary perfusion scintigraphy in predicting postoperative pulmonary function after lung cancer resection. DDR accurately estimated postoperative FEV_1_, DLco values, and identified patients at risk for respiratory complications, supporting its use as a simple, low‐cost alternative to conventional scintigraphy for preoperative assessment [25].

Lastly, Santibanez et al. integrated DDR with machine learning to derive dynamic pulmonary function test (dPFT) metrics that strongly correlate (*r* > 0.5) with conventional pulmonary function test volumes, suggesting DDR as a viable alternative in settings lacking traditional PFT access [26].

## 7 Pulmonary Circulation and Disorders

**FIGURE 4:**
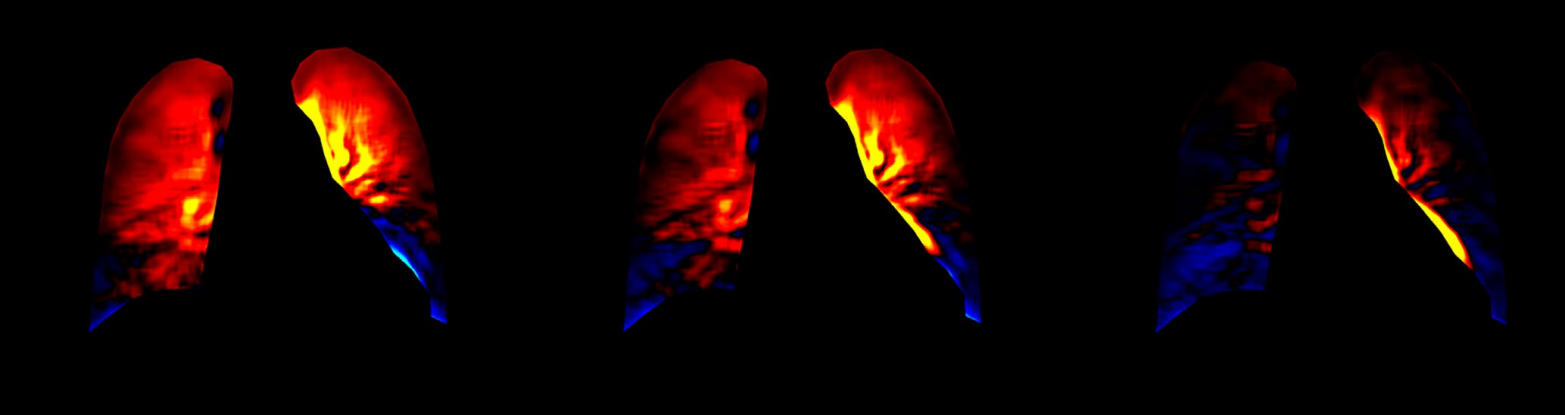
Assessment of signal value change associated with pulmonary function utilising PH2‐Mode from a DDR acquisition. (Images provided by Minolta Healthcare Americas, used with permission. Obtained from a mobile DDR system in an ICU.)

DDR's applicability extends to pulmonary vascular imaging through integration with advanced post‐processing tools like PH2‐Mode, Figure 4, which visualises signal value changes indicative of pulmonary circulation patterns [27]. Preliminary studies demonstrate that DDR can provide dynamic pulmonary blood flow information comparable to established nuclear medicine techniques such as scintigraphy and ventilation‐perfusion (V/Q) scans, with the added advantages of lower cost and reduced radiation exposure [27, 28, 29, 30]. For example, DDR has been shown to effectively assess conditions including partial anomalous pulmonary venous connection and thromboembolic pulmonary hypertension (CTEPH), offering diagnostic accuracy similar to traditional methods but with improved accessibility [27, 29]. While promising, these applications currently rely on limited patient cohorts, underscoring the need for larger studies to validate DDR's clinical utility in pulmonary vascular assessment.

## 8 Lung Tumour Tracking

In oncological settings, DDR's high temporal resolution imaging supports real‐time tracking of lung tumour motion during radiotherapy, a critical component in ensuring accurate radiation delivery and minimising damage to surrounding healthy tissue. Studies employing DDR at frame rates up to 15 fps demonstrate strong correlation with four‐dimensional CT (4D‐CT) for tumour movements along superior–inferior and left–right axes, though tracking anterior–posterior motion remains more challenging [31]. This capability positions DDR as a potentially valuable tool for tumour motion management, particularly in adaptive radiotherapy protocols; although further technological refinement and validation are necessary.

## 9 Cardiac Applications

Emerging research suggests DDR may serve as a minimally invasive alternative for hemodynamic assessment in patients with heart failure. DDR‐derived pixel intensity metrics have been correlated strongly with invasive arterial pressure measurements obtained via right heart catheterization, indicating that DDR can capture meaningful hemodynamic changes non‐invasively [32]. Imaging protocols typically involve breath holds lasting several seconds at frame rates around 15 fps, balancing temporal resolution with patient comfort. Despite these encouraging findings, current studies are limited by small sample sizes and focus primarily on severe heart failure cases, highlighting the need for broader research to establish DDR's clinical role in cardiac diagnostics.

## 10 Orthopaedic Applications

DDR's ability to provide low‐dose, dynamic imaging of musculoskeletal structures offers substantial benefits in orthopaedic assessment. Clinical applications include evaluating joint kinematics post‐surgery, tracking recovery following cervical spine injuries, and analysing joint movements during functional activities such as gait [33, 34, 35, 36]. For example, DDR has been used to document shoulder instability and to quantitatively assess shoulder abduction, providing objective data to guide rehabilitation [33, 35]. Compared to fluoroscopy, DDR reduces radiation exposure and costs while maintaining sufficient image quality and temporal resolution to inform clinical decision‐making [35]. These advantages underscore DDR's potential to become an integral tool in orthopaedic diagnostics and functional assessment.

## 11 Current Limitations

Various studies have outlined the promising applications of DDR, but notable limitations currently hinder its widespread clinical adoption.

The first significant limitation is the lack of clinical implementation. While DDR shows potential in various medical imaging scenarios, much of the research is still preliminary, focusing on theorising or exploring DDR's integration into clinical settings on a small scale/cohort. This is outlined in studies like Uchida et al., Fyles et al. and Hoshino et al. where their studies included results based on only one patient. These small cohorts indicate that more extensive research, including clinical trials and longitudinal studies, is necessary to fully assess DDR's usability, effectiveness and potential benefits in real‐world clinical environments.

The second limitation is DDR's ability to replace existing imaging modalities. While DDR can produce results comparable to those of established technologies like X‐ray, CT and fluoroscopy, it often fails to surpass these modalities regarding image quality or diagnostic accuracy. This is highlighted in studies like Yamasaki et al. and Kitamura et al., where while DDR might offer a possible alternative for diagnostic imaging, it doesn't necessarily perform or exceed the current modalities used. As a result, clinics may not see a compelling reason to invest in DDR systems, especially when they already have reliable imaging equipment and trained staff familiar with those existing technologies.

Additionally, there is the lack of clarity regarding whether the studies reviewed utilised stationary or mobile DDR systems. While the initial aim was to explore the applications of both device types, many studies did not specify the equipment used, making it difficult to draw clear conclusions about the comparative performance, feasibility, or clinical utility of mobile versus fixed DDR systems. This ambiguity limits the ability to assess mobile DDR's specific advantages—such as point‐of‐care use in critical care settings, which may be a key factor in its broader adoption.

Finally, in studies like Shibuya et al. and Watanabe et al., where DDR is being looked at as an alternative modality to non‐ionising diagnostic methods, it might be problematic for a clinic to justify its implementation over methods like ultrasound due to the patients' increased radiation exposure.

DDR's versatility allows it to be applied across a broad range of imaging needs. This general competence, while helpful, may limit DDR's justification for implementation, as healthcare facilities may prefer specialised imaging technologies that excel in specific diagnostic tasks. However, as outlined in this review, there is growing interest in DDR's use as a portable device, particularly for chest X‐rays, where its low radiation dose, post processing software and dynamic imaging capabilities could offer distinct advantages over other modalities.

## 12 Future Studies

To further advance the application of DDR, it is essential to conduct clinical trials comparing DDR with traditional imaging techniques like CT and fluoroscopy to evaluate its diagnostic accuracy and reliability across a variety of clinical scenarios. These studies should include diverse patient populations to assess the modality's effectiveness in both common and rare respiratory and cardiac conditions. Longitudinal studies are also needed to track the progression of diseases, such as pulmonary fibrosis or diaphragm dysfunction, to determine how DDR can be used in disease monitoring and management. Such studies will help establish the role of DDR in providing real‐time insights into disease evolution and response to treatment, further solidifying its potential in clinical practice.

Another important avenue for future research involves the analysis of the portable aspect of DDR devices, which could greatly expand access to this technology, particularly in settings where traditional imaging methods are impractical. These devices could be valuable for point‐of‐care assessments, especially in rural or underserved areas, offering a non‐invasive and low‐radiation alternative to other imaging methods. To support this shift, additional research into the cost‐effectiveness and logistical feasibility of implementing portable DDR may be beneficial. Evaluating the economic and practical implications of DDR, including its impact on patient outcomes and healthcare costs, will be critical to its widespread adoption.

## 13 Conclusion

This literature review has examined DDR, a technique that enables visualisation of anatomical motion through rapid sequences of X‐ray images captured at varying frame rates. DDR demonstrates promising clinical potential across a range of applications, including the diagnosis of acute pulmonary embolism, assessment of pulmonary circulation, and possible uses in chest, cardiac and orthopaedic imaging. Notable advantages of DDR include its portability and the integration of advanced post‐processing software, such as DI‐X1, which enhances its diagnostic utility by enabling clinicians to refine and track dynamic images.

Despite these strengths, a central consideration in the literature is whether DDR offers sufficient benefits over established imaging modalities like CT, standard X‐ray and fluoroscopy. Current research is limited by small patient cohorts, making it difficult to fully validate DDR's clinical accuracy, reliability and cost‐effectiveness. As such, further large‐scale studies are necessary to determine the modality's true value and potential role within routine clinical practice.

This review extends beyond the technical scope of previous studies by addressing DDR's practical adoption in clinical settings. In doing so, it underscores the modality's growing relevance and its potential to advance diagnostic imaging practice.

## Conflicts of Interest

The authors declare no conflicts of interest.

## Data Availability

The data that support the findings of this study are available from the corresponding author upon reasonable request.

## References

[1] M. Cè , G. Oliva , F. L. Rabaiotti , et al., “Portable Dynamic Chest Radiography: Literature Review and Potential Bedside Applications,” Medical Science 12, no. 1 (2024): 10, 10.3390/medsci12010010. PMC10885043 38390860

[2] F. Fyles , T. S. FitzMaurice , R. E. Robinson , R. Bedi , H. Burhan , and M. J. Walshaw , “Dynamic Chest Radiography: A State‐of‐the‐Art Review,” Insights Into Imaging 14, no. 1 (2023): 107, 10.1186/s13244-023-01451-4. 37332064 PMC10277270

[3] J. Isaacson , “Digital Radiography (DR),” (2022), https://www.itnonline.com/content/blogs/jenelle‐isaacson‐contributing‐editor/blog‐assessing‐anatomy‐motion‐dynamic‐digital.

[4] “Dynamic Digital X‐Ray,” https://www.cmsimaging.com/ddr.html.

[5] “Good Practices in Interventional Procedures,” (2017), https://www.iaea.org/resources/rpop/health‐professionals/interventional‐procedures/good‐practices‐in‐interventional‐fluoroscopy.

[6] D. L. Smith , J. P. Heldt , G. D. Richards , et al., “Radiation Exposure During Continuous and Pulsed Fluoroscopy,” Journal of Endourology 27, no. 3 (2013): 384–388, 10.1089/end.2012.0213. 22966826

[7] S. Singh , “Dynamic Digital Radiography in Chest Imaging,” (2021), https://www.youtube.com/watch?v=QE1ruslKhVU.

[8] “Dynamic Digital Radiography,” https://healthcare.konicaminolta.us/radiography/dynamic‐digital‐radiography.

[9] T. Hida , Y. Yamada , M. Ueyama , et al., “Time‐Resolved Quantitative Evaluation of Diaphragmatic Motion During Forced Breathing in a Health Screening Cohort in a Standing Position: Dynamic Chest Phrenicography,” European Journal of Radiology 113 (2019): 59–65, 10.1016/j.ejrad.2019.01.034. 30927960

[10] T. FitzMaurice , C. McCann , D. Nazareth , and M. Walshaw , “P237 Dynamic Chest Radiography: A Novel Tool for the Assessment of Diaphragm Palsy,” in Time for Sleep (BMJ Publishing Group Ltd and British Thoracic Society, 2021), A217.1–A217, 10.1136/thorax-2020-BTSabstracts.381.

[11] S. Uchida , T. Matsunaga , A. Hattori , M. Fukui , K. Takamochi , and K. Suzuki , “Efficacy of Dynamic Chest Radiography for Evaluating Surgical Treatment of Diaphragmatic Paralysis,” Annals of Thoracic Surgery Short Reports 2, no. 3 (2024): 502–505, 10.1016/j.atssr.2024.03.007. 39790427 PMC11708631

[12] T. FitzMaurice , C. McCann , D. S. Nazareth , and M. J. Walshaw , “Characterisation of Hemidiaphragm Dysfunction Using Dynamic Chest Radiography: A Pilot Study,” ERJ Open Research 8, no. 1 (2022): 00343–02021, 10.1183/23120541.00343-2021. 35211619 PMC8862633

[13] Y. Shibuya , K. Hirano , H. Machida , et al., “Bilateral Recurrent Laryngeal Nerve Paralysis Diagnosed Using Dynamic Digital Radiography During the COVID‐19 Pandemic,” Clinical Case Reports 10, no. 7 (2022): e6124, 10.1002/ccr3.6124. 35898737 PMC9309747

[14] Y. Koyama , Y. M. R A , T. O. Slp , M. Toyokura , K. Mizuno , and Y. Masakado , “Bedside Diagnosis of Silent Aspiration Using Mobile Dynamic Digital Radiography: A Preliminary Study,” European Archives of Oto‐Rhino‐Laryngology 281 (2024): 5527–5533, 10.1007/s00405-024-08785-9. 38976064 PMC11416413

[15] F. Fyles , A. Nuttall , T. FitzMaurice , R. Robinson , and H. Burhan , “Identification of Large Airway Collapse With Symptoms Using Dynamic Chest Radiography,” American Journal of Respiratory and Critical Care Medicine 207, no. 4 (2023): 485–486, 10.1164/rccm.202206-1131IM. 36548808

[16] N. Ohkura , R. Tanaka , S. Watanabe , et al., “Chest Dynamic‐Ventilatory Digital Radiography in Chronic Obstructive or Restrictive Lung Disease,” International Journal of Chronic Obstructive Pulmonary Disease 16 (2021): 1393–1399, https://www.dovepress.com/chest‐dynamic‐ventilatory‐digital‐radiography‐in‐chronic‐obstructive‐o‐peer‐reviewed‐fulltext‐article‐COPD. 34040366 10.2147/COPD.S309960 PMC8140888

[17] N. Ohkura , R. Tanaka , S. Watanabe , et al., “Investigation of Quantitative Evaluation of Pulmonary Function by Dynamic Digital Radiography,” in B80‐1. Methodological Advancements in Pulmonary Imaging (American Thoracic Society, 2024), A4512, 10.1164/ajrccm-conference.2024.209.1_MeetingAbstracts.A4512.

[18] Y. Kitahara , R. Tanaka , H. R. Roth , et al., “Lung Segmentation Based on a Deep Learning Approach for Dynamic Chest Radiography,” in Medical Imaging 2019: Computer‐Aided Diagnosis, ed. H. K. Hahn and K. Mori (SPIE, 2019), 130, 10.1117/12.2512711.

[19] N. Ishihara , R. Tanaka , W. P. Segars , E. Abadi , and E. Samei , “Estimation of Lung Volume Changes From Frontal and Lateral Views of Dynamic Chest Radiography Using a Convolutional Neural Network Model: A Computational Phantom Study,” in Medical Imaging 2021: Physics of Medical Imaging, ed. H. Bosmans , W. Zhao , and L. Yu (SPIE, 2021), 179, 10.1117/12.2579948.

[20] T. Watanabe , E. Suzuki , N. Yoshii , et al., “Preoperative Detection of Pleural Adhesions Using Dynamic Chest Radiography: Prospective Analysis,” Journal of Thoracic Disease 15, no. 3 (2023): 1096–1105, 10.21037/jtd-22-1226. 37065574 PMC10089839

[21] T. Nikaido , Y. Tanino , Y. Sato , et al., “Two Cases of Idiopathic Pulmonary Fibrosis Evaluated by Dynamic Digital Radiography for Diaphragmatic Motion and Disease Progression,” Respirology Case Reports 12, no. 2 (2024): e01301, 10.1002/rcr2.1301. 38384743 PMC10880408

[22] M. Ueyama , S. Hashimoto , A. Takeda , et al., “Prediction of Forced Vital Capacity With Dynamic Chest Radiography in Interstitial Lung Disease,” European Journal of Radiology 142 (2021): 109866, 10.1016/j.ejrad.2021.109866. 34365304

[23] R. Tanaka , I. Matsumoto , M. Tamura , et al., “Dynamic Chest Radiography: Clinical Validation of Ventilation and Perfusion Metrics Derived From Changes in Radiographic Lung Density Compared to Nuclear Medicine Imaging,” Quantitative Imaging in Medicine and Surgery 11, no. 9 (2021): 4016–4027, 10.21037/qims-20-1217. 34476186 PMC8339647

[24] N. Ohkura , K. Kasahara , S. Watanabe , et al., “Dynamic‐Ventilatory Digital Radiography in Air Flow Limitation: A Change in Lung Area Reflects Air Trapping,” Respiration 99, no. 5 (2020): 382–388, 10.1159/000506881. 32348982 PMC7845443

[25] J. Hanaoka , M. Yoden , K. Hayashi , et al., “Dynamic Perfusion Digital Radiography for Predicting Pulmonary Function After Lung Cancer Resection,” World Journal of Surgical Oncology 19 (2021): 43, 10.1186/s12957-021-02158-w. 33563295 PMC7874664

[26] V. Santibanez , T. J. Pisano , F. X. Doo , et al., “Dynamic Digital Radiography Pulmonary Function Testing,” CHEST Pulmonary 2, no. 3 (2024): 100052, 10.1016/j.chpulm.2024.100052. PMC13418388 42548400

[27] S. Hoshino , H. Miyatake , and Y. Maruo , “Using Dynamic Digital Radiography to Assess Pulmonary Circulation Imaging in a Patient With Congenital Heart Disease,” International Journal of Cardiovascular Imaging 38, no. 6 (2021): 1179–1180, 10.1007/s10554-021-02517-4. 34971419

[28] D. Toyomura , K. Yamamura , and Y. Yamasaki , “Dynamic Digital Radiography: A Novel Quantitative Modality to Assess the Pulmonary Blood Flow,” European Heart Journal 44, no. 16 (2023): 1479, 10.1093/eurheartj/ehad112. 36883345

[29] Y. Yamasaki , K. Abe , T. Kamitani , et al., “Efficacy of Dynamic Chest Radiography for Chronic Thromboembolic Pulmonary Hypertension,” Radiology 306, no. 3 (2023): e220908, 10.1148/radiol.220908. 36346313

[30] Y. Yamasaki , T. Kamitani , K. Sagiyama , et al., “Dynamic Chest Radiography for Pulmonary Vascular Diseases: Clinical Applications and Correlation With Other Imaging Modalities,” Japanese Journal of Radiology 42, no. 2 (2024): 126–144, 10.1007/s11604-023-01483-2. 37626168 PMC10811043

[31] K. Kitamura , K. Takayama , R. Yamazaki , Y. Ueda , and S. Nishiki , “A New Method for Assessing Lung Tumor Motion in Radiotherapy Using Dynamic Chest Radiography,” Journal of Applied Clinical Medical Physics 23, no. 10 (2022): e13736, 10.1002/acm2.13736. 35930373 PMC9588259

[32] H. Hiraiwa , G. Sakamoto , R. Ito , et al., “Dynamic Chest Radiography as a Novel Minimally Invasive Hemodynamic Imaging Method in Patients With Heart Failure,” European Journal of Radiology 161 (2023): 110729, 10.1016/j.ejrad.2023.110729. 36804311

[33] Z. B. Hussain , S. R. Khawaja , A. L. Karzon , A. S. Ahmed , M. B. Gottschalk , and E. R. Wagner , “Digital Dynamic Radiography—A Novel Diagnostic Technique for Posterior Shoulder Instability: A Case Report,” JSES International 7 (2023): 523–526, 10.1016/j.jseint.2023.02.015. 37426924 PMC10328772

[34] A. R. Gezici , Y. Dagistan , S. E. Cancan , et al., “Upper Cervical Spinal Injuries in Elderly Patients: Age‐Specific Treatment,” Biomedical Research 28, no. 2 (2017): 778–785.

[35] K. Sakuda , S. Sanada , R. Tanaka , K. Kitaoka , N. Hayashi , and Y. Matsuura , “Functional Shoulder Radiography With Use of a Dynamic Flat Panel Detector,” Radiological Physics and Technology 7, no. 2 (2014): 254–261, 10.1007/s12194-014-0257-2. 24515244 PMC4097328

[36] H. A. Gray , S. Guan , L. T. Thomeer , A. G. Schache , R. De Steiger , and M. G. Pandy , “Three‐Dimensional Motion of the Knee‐Joint Complex During Normal Walking Revealed by Mobile Biplane x‐Ray Imaging,” Journal of Orthopaedic Research 37, no. 3 (2019): 615–630, 10.1002/jor.24226. 30680795

[37] W. Huda , G. A. Sandison , R. F. Palser , and D. Savoie , “Radiation Doses and Detriment From Chest X‐Ray Examinations,” Physics in Medicine and Biology 34, no. 10 (1989): 1477–1492, 10.1088/0031-9155/34/10/010. 2813514

[38] R. Smith‐Bindman , “Radiation Dose Associated With Common Computed Tomography Examinations and the Associated Lifetime Attributable Risk of Cancer,” Archives of Internal Medicine 169, no. 22 (2009): 2078, 10.1001/archinternmed.2009.427. 20008690 PMC4635397

